# Artificial intelligence for the detection and classification of occupational pneumoconioses: a systematic review

**DOI:** 10.47626/1679-4435-2026-1529

**Published:** 2026-10-20

**Authors:** Ashley Delgado-Cano, Andy Torres, Diemen Delgado-García

**Affiliations:** 1 Universidad Andrés Bello, School of Medicine, Viña del Mar, Valparaíso, Chile; 2 The University of Texas Rio Grande Valley, Department of Psychological Science, Edinburg, Texas, USA; 3 Harvard Medical School, McLean Hospital, Behavioral Health Partial Hospital Program, Belmont, Massachusetts, USA; 4 University of Texas Rio Grande Valley, School of Medicine, Edinburg, Texas, USA; 5 Universidad de Los Andes, Department of Epidemiology and Health Studies, Las Condes, Santiago, Chile; 6 Occupational Disease Qualification Committee, Asociación Chilena de Seguridad, Providencia, Chile

**Keywords:** artificial intelligence, occupational pneumoconioses, chest radiography, deep learning, occupational health

## Abstract

Occupational pneumoconioses remain an important occupational health problem, and chest imaging interpretation may show substantial interobserver variability. Artificial intelligence has attracted increasing interest as a supportive tool for the detection, classification, and staging of these diseases. This systematic review aimed to synthesize and critically evaluate the available evidence on the use of artificial intelligence for imaging-based assessment of occupational pneumoconioses. PubMed, Scopus, Web of Science Core Collection, and IEEE Xplore were searched for studies published from January 2015 through June 2025, in accordance with the Preferred Reporting Items for Systematic Reviews and Meta-Analyses 2020 guidelines. Of 486 identified records, 137 duplicates were removed, 62 articles underwent full-text assessment, and 10 studies met the eligibility criteria. All studies used chest radiography, were published between 2020 and 2024, and were conducted in China and Australia. Convolutional neural networks were the predominant approach, with a shift toward ensemble models, multistage strategies, and attention mechanisms. The studies reported favorable performance but were heterogeneous in populations, diagnostic tasks, reference standards, and validation strategies. The available evidence does not allow deriving an overall estimate of diagnostic performance or establishing a consistent superiority of AI over radiologists. Artificial intelligence shows potential as a complementary tool for occupational surveillance and radiographic assessment; however, external, prospective, and multicenter validation is required before clinical and occupational implementation.

## INTRODUCTION

Occupational pneumoconioses are a group of pulmonary diseases caused by inhalation and retention of mineral dusts and other inorganic particles, predominantly due to occupational exposure [^1^]. They include silicosis, coal workers’ pneumoconiosis, and other forms associated with different mineral agents. These conditions may lead to progressive parenchymal changes and pulmonary fibrosis, and, in advanced stages, may result in progressive massive fibrosis [^2^]. Pneumoconioses remain an important occupational health problem in activities involving exposure to mineral dust and respirable crystalline silica, such as mining, quarrying, construction, sandblasting, certain foundry operations, and natural and engineered stone processing [^3^].

Pneumoconioses may be associated with disability, impaired quality of life, and loss of work capacity, imposing a substantial social and economic burden on workers and their families, as well as on health care and social security systems [^4^]. The continued occurrence of pneumoconiosis among exposed workers despite the implementation of exposure control measures and surveillance programs highlights the need to strengthen prevention, early detection, and follow-up strategies for workers at risk [^5^].

The diagnosis and surveillance of pneumoconioses rely on occupational history, clinical assessment, and imaging. The International Labor Organization (ILO) Classification of Radiographs of Pneumoconioses provides a standardized method for systematically describing and recording radiographic abnormalities associated with pneumoconioses. Small opacities are classified according to their profusion, shape, size, and distribution across lung zones [^6^]. However, radiographic interpretation may be subject to interobserver variability and depends on the reader’s experience and specialty training, particularly when assessing the profusion and shape of small opacities and in difficult-to-classify cases. Coexisting pulmonary abnormalities may also complicate the radiographic differentiation of pneumoconioses [^7^]. Computed tomography, including high-resolution computed tomography, provides more detailed and sensitive characterization of pulmonary abnormalities than chest radiography, particularly in early-stage disease. However, its widespread implementation as an occupational surveillance tool is limited by factors such as availability, cost, specialized infrastructure requirements, and radiation exposure [^8^].

In this context, artificial intelligence (AI) methods, particularly those based on machine learning and deep learning, have gained increasing attention for automated medical image analysis, including detection, classification, and segmentation tasks [^9^]. Convolutional neural networks (CNNs), including architectures such as ResNet and DenseNet, along with segmentation models, attention mechanisms, and other deep learning approaches, can identify complex imaging patterns and automate a range of detection, classification, and staging tasks [^10^]. These approaches have been applied to the radiographic detection, classification, and staging of occupational pneumoconioses. However, the available studies show substantial differences in the populations evaluated, types of pneumoconiosis, exposure histories, outcome definitions, model architectures, and validation strategies.

This heterogeneity is critical to interpreting the evidence, since an AI model developed to detect or classify a particular pneumoconiosis in a specific population cannot automatically be assumed to generalize to other populations, diseases, occupational exposures, or clinical settings without external validation in independent settings [^11^]. Likewise, performance metrics derived from different diagnostic tests, populations, reference standards, and validation designs may not be directly comparable and should be interpreted in light of the methodological and clinical characteristics of each study [^12^]. Therefore, assessments of AI performance should encompass not only diagnostic performance metrics but also model generalizability.

Model generalizability may be affected by differences in the demographic and occupational characteristics of the populations, image acquisition equipment, radiographic protocols, image quality, classification criteria, and reference standards. These factors can lead to shifts in data distribution (data shift or distribution shift) and reduce model performance when models are applied to populations, centers, image acquisition equipment, or clinical settings that differ from those in which the models were developed [^13^]. Therefore, the lack of sufficient external validation, multicenter studies, and prospective evaluations represents a major barrier to establishing the clinical and occupational utility of these technologies. Moreover, overlifting and limited generalizability, together with insufficient transparency, limited reproducibility, and potential algorithmic biases, may compromise the applicability of these technologies to clinical settings and populations different from those used during their development [^14^].

Methodological assessment is essential for the appropriate interpretation of these results, since tools such as the Prediction model Risk Of Bias ASsessment Tool (PROBAST) and its AI-specific update, PROBAST+AI, provide a structured approach to assessing methodological quality, risk of bias, and concerns regarding the applicability of prediction models [^15^]. In turn, the Transparent Reporting of a multivariable prediction model for Individual Prognosis Or Diagnosis (TRIPOD) initiative provides a framework for assessing the transparency and completeness of reporting, whereas its update, TRIPOD+AI, incorporates specific recommendations for studies of prediction models that use AI and machine learning methods [^16^]. The use of these tools can help identify limitations in participant selection, predictor and outcome definitions, analysis, validation, and reproducibility.

From an occupational health perspective, AI could support automated screening, prioritize radiographs for specialist review, and facilitate the analysis of large amounts of images. However, the implementation of these technologies should not rely exclusively on high performance metrics obtained from internal datasets. The utility of an AI system requires evidence of reproducibility, external validation, representativeness of the target populations, feasibility of implementation, and robust performance under conditions that approximate real-world clinical and occupational practice [^17^]. In this context, AI should be regarded as a potentially complementary tool for specialist assessment rather than a substitute for clinical and radiological judgment.

Consequently, this systematic review aimed to synthesize and critically evaluate the available evidence on the use of AI for the imaging-based detection, classification, and staging of occupational pneumoconioses. Specifically, the review sought to: (1) characterize the populations, diseases, occupational exposures, imaging modalities, and AI algorithms investigated; (2) synthesize diagnostic performance metrics, including sensitivity, specificity, accuracy, and area under the curve (AUC), when available; (3) examine the evidence derive from comparisons between AI and human readers; and (4) asses the main methodological limitations, potential sources of bias, validation strategies, and evidence of external generalizability, with particular emphasis on the potential application of these technologies in occupational health surveillance programs.

## METHODS

### REVIEW DESIGN AND GUIDELINES

A systematic review was conducted in accordance with the Preferred Reporting Items for Systematic Reviews and Meta-Analyses (PRISMA) 2020 guidelines to ensure transparent, complete, and reproducible reporting of evidence identification, selection, assessment, and synthesis [^18^]. The eligibility criteria, study selection and data extraction procedures, methodological assessment, and synthesis strategy were predefined.

### SEARCH STRATEGY

Systematic searches were conducted in PubMed/MEDLINE, Scopus, Web of Science Core Collection, and IEEE Xplore to identify publications from January 1, 2015, through June 30, 2025. The final electronic search was completed on June 30, 2025. The search was limited to English-language publications within the specified period.

Controlled vocabulary terms, when available, were combined with keywords related to pneumoconiosis, silicosis, AI, machine learning, deep learning, radiomics, computer-aided detection, and CNNs. Boolean operators and database-specific syntax were used.

A representative strategy for PubMed was as follows: (“silicosis” OR “pneumoconiosis”) AND (“artificial intelligence” OR “machine learning” OR “deep learning” OR “radiomics” OR “convolutional neural network” OR “computer-aided detection”). The searches were independently verified by two reviewers. Furthermore, the reference lists of potentially eligible articles were screened to identify additional studies.

### ELIGIBILITY CRITERIA

Studies were included if they met the following criteria:

1. **Population and disease:** workers or individuals with occupational pneumoconiosis or a history of occupational exposure to mineral dust, including participants with pneumoconiosis or exposed workers evaluated by chest radiography for screening, surveillance, detection, diagnosis, classification, or staging.
2. **Analysis method:** application of AI methods, including machine learning, deep learning, CNNs, ResNet or DenseNet-derived architectures, segmentation, attention mechanisms, ensemble learning, or other computational methods designed to analyze images of occupational pneumoconioses.
3. **Imaging modality:** use of chest radiography, including digital radiography, for model development, evaluation, or validation.
4. **Outcomes:** reports of at least one performance metric, such as AUC, sensitivity, specificity, accuracy, precision, recall, F1 score, quadratic weighted kappa (QWK), or other metrics relevant to the task evaluated.
5. **Stuy design:** original retrospective or prospective studies published in peer-reviewed journals that used imaging databases for the development, evaluation, or validation of AI-based models.

When a study included different forms of pneumoconiosis, the specific disease, population, and diagnostic task actually evaluate were considered. Equivalence between different types of pneumoconiosis was not assumed, nor were results extrapolated across populations or diseases.

The ILO International Classification of Radiographs of Pneumoconioses was considered a relevant radiological reference standard when used in the included studies [^6^], although its use was not required for inclusion. Other well-defined reference standards were also accepted, such as expert consensus and specialized radiological assessments.

### EXCLUSION CRITERIA

Exclusion criteria were as follows:

- animal studies;
- case reports and case series;
- reviews, editorials, comments, and opinion articles;
- conference abstracts without available full text;
- studies without performance metrics or sufficient results to assess method performance;
- studies focused exclusively on algorithm development without imaging-based assessment;
- studies unrelated to occupational pneumoconiosis or to relevant occupational populations or exposures;
- publications outside the specified period.

### STUDY SELECTION

Two reviewers (DDG and ADC) independently screened titles and abstracts and subsequently assessed the full texts of potentially eligible articles according to the predefined criteria. Disagreements were resolved through discussion and consensus and, when necessary, by consultation with a third reviewer.

The search identified 486 records, of which 137 were removed as duplicates. The remaining 349 records underwent title and abstract screening. Subsequently, 62 articles were assessed in full text, and 10 studies ultimately met the inclusion criteria.

### DATA EXTRACTION

A standardized form was used to independently extract the following variables:

- author, year, and country;
- type of pneumoconiosis and population characteristics;
- occupational exposure, when available;
- dataset size and distribution;
- imaging modality;
- diagnostic test;
- AI architecture or method;
- training strategy;
- validation strategy;
- reference standard;
- performance metrics;
- comparison with human readers, when available;
- main findings and limitations.

Where possible, validation strategies were categorized internal validation, cross-validation, evaluation on an independent test set, external validation, or prospective evaluation.

Performance metrics were recorded according to the values reported in the original publications, and a metric was unavailable, no imputation or extrapolation was performed using data from other studies.

Derived values were calculated only when they could be reproducibly determined from the published data and were clearly distinguished from the metrics originally reported.

### RISK OF BIAS AND REPORTING QUALITY ASSESSMENT

Two reviewers independently assessed risk of bias and concerns regarding applicability using PROBAST, across the participant, predictor, outcome, and analysis domains [^15^]. Each domain was classified according to the criteria specified by the tool, and discrepancies were resolved through consensus.

Reporting quality and transparency were assessed using the TRIPOD checklist. The assessment examined the reporting of participants and datasets, predictors and outcomes, model development, data handling, performance measures, and validation procedures [^16^].

### DATA SYNTHESIS

Given the heterogeneity in populations, types of pneumoconiosis, diagnostic tasks, AI architectures, reference standards, and validation strategies, a quantitative meta-analysis was not performed. Instead, a structured narrative synthesis was conducted.

Studies were organized according to disease, population and occupational exposure, diagnostic task, AI methodology, and validation strategy. Performance metrics were presented individually, and no pooled estimates calculated when methodological differences precluded direct comparison.

When available, comparisons between AI and human readers were described according to population, diagnostic task, reference standard, and evaluation design. Metrics from different studies were not combined when they were not directly comparables.

A clear distinction was made between internal validation, cross-validation, evaluation on an independent test set, external validation, prospective evaluation. In particular, evaluation on an independent test set was not automatically considered equivalent to multicenter external validation.

## RESULTS

### STUDY SELECTION

The initial search identified 486 records across the four databases, of which 137 were removed as duplicates. The remaining 349 records underwent title and abstract screening, after which the full texts of 62 articles were assessed according to the predefined eligibility criteria. Following final verification of bibliographic identity, eligibility, and correspondence with the data extraction records, 10 studies were included in the qualitative synthesis. The study identification, selection, and inclusion process is shown in the PRISMA flow diagram (Figure 1).

**Figure 1.**
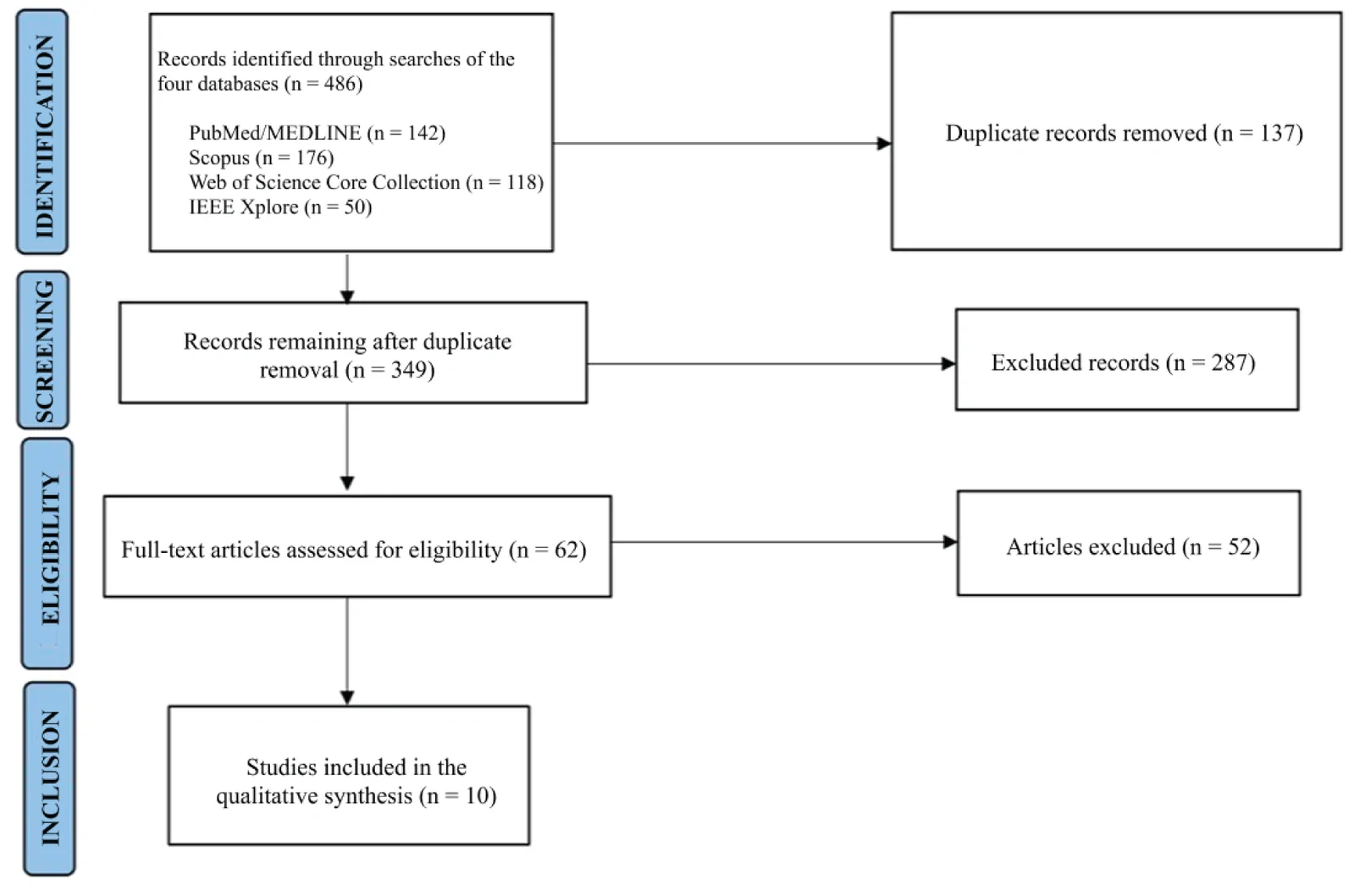
Preferred Reporting Items for Systematic Reviews and Meta-Analyses 2020 flow diagram of study identification, screening, eligibility assessment, and inclusion.

The included studies were those by Wang et al. [^19^], Zhang et al. [^20^], Yang et al. [^21^], Devnath et al. [^22^], Dong et al. [^23^], Devnath et al. [^24^], Sun et al. [^25^], Liu et al. [^26^], Li et al. [^27^], and Wang et al. [^28^].

All 10 studies evaluated AI applications for the detection, classification, or staging of occupational pneumoconiosis using chest radiography.

### GENERAL CHARACTERISTICS OF THE INCLUDED STUDIES

The studies were published entre 2020 y 2024 and were conducted exclusively in China (n = 8) and Australia (n = 2). All studies used chest radiography, predominantly digital radiography, although they differed in the population studied, type of pneumoconiosis, dataset sizes, diagnostic tests, and validation strategies.

Wang et al. analyzed 1,881 digital radiographs from 923 cases of pneumoconiosis and 958 individuals without pneumoconiosis, all of whom had a history of dust exposure [^19^]. Zhang et al. included a training cohort of 805 participants and an independent test cohort of 411 participants, comprising controls and patients with stage I–III pneumoconiosis [^20^]. Yang et al. analyzed 1,760 posteroanterior (PA) digital chest radiographs from 1,248 individuals with pneumoconiosis and 512 without pneumoconiosis [^21^].

Devnath et al. assessed 153 PA chest radiographs, including 71 with parenchymal opacities consistent with pneumoconiosis and 82 without pneumoconiosis [^22^], Dong et al. included 217 chest radiographs from 149 patients with coal workers’ pneumoconiosis and 68 dust-exposed workers [^23^]. Devnath et al. analyzed 153 PA chest radiographs, of which 71 corresponded to cases of pneumoconiosis and 82 to individuals without pneumoconiosis [^24^].

Sun et al. evaluated chest radiographs from patients with pneumoconiosis to develop a deep learning model for disease staging into four categories: normal, stage I, stage II, and stage III [^25^]. The dataset comprised 1,372 radiographs corresponding to 845 normal individuals, 293 stage I cases, 132 stage II cases, and 102 stage III cases [^25^]. Liu et al. analyzed 498 chest radiographs, corresponding to 193 normal individuals and patients with stage I (n = 147), stage II (n = 108), and stage III (n = 50) pneumoconiosis to develop a deep learning systema for disease diagnosis and staging [^26^].

Li et al. used a database of 49,872 labeled chest radiographs, including 24,437 cases of pneumoconiosis and 25,435 individuals without pneumoconiosis [^27^]. Model performance was subsequently evaluated on an independent set of 495 radiographs, comprising 261 positive cases and 234 negative ones [^27^].

Wang et al. developed and evaluated a Multi-scale Lesion-aware Attention Network (MLANet) model using digital radiographs from two centers, with training, internal testing, and external datasets, as well as prospective evaluation [^28^]. The latter comprised 87 radiographs and enabled comparison of model performance with that of qualified radiologists [^28^].

### ARTIFICIAL INTELLIGENCE ARCHITECTURES

CNNs were the predominant approach, encompassing architectures and strategies such as Inception-V3, ResNet-34, ResNet-50, DenseNet, EfficientNet, CheXNet, U-Net, ShuffleNet V2, Efficient Attention Channel (ECA)-Net, ensemble models, and attention-based architectures.

Wang et al. used Inception-V3 [^19^]. Zhang et al. combined lung segmentation and a CNN model for screening and staging [^20^], whereas Yang et al. employed U-Net and ResNet-34 [^21^].

Devnath et al. combined CheXNet and deep features with support vector machine and CNN classifiers [^22^]. In another study, Devnath et al. developed an ensemble model integrating CheXNet, DenseNet, InceptionV3, Xception, and ResNet-50 [^24^].

Dong et al. used ShuffleNet V2 and ECA-Net with data augmentation [^23^]. Sun et al. developed an Asymmetric EncoderDecoder Network (AED-Net) [^25^], whereas Liu et al. employed EfficientNet and a multi-stage model based on ResNet-34 and U-Net [^26^].

Li et al. applied a deep CNN based on ResNet-101 [^27^]. Wang et al. developed a MLANet model using Res2Net-50 as its backbone for diagnosis and staging [^28^].

Overall, the studies covered a broad range of approaches, ranging from conventional CNNs and feature extraction methods to ensemble strategies, multistage models, and attention-based architectures.

### DIAGNOSTIC PERFORMANCE

The included studies reported favorable performance metrics across different diagnostic tasks and evaluation designs. Because of this heterogeneity, pooled performance estimates were not calculated.

Using Inception-V3, Wang et al. reported an AUC of 0.878 (95% CI, 0.811-0.946) [^19^]. Zhang et al. obtained an accuracy of 0.973, sensitivity of 0.981, and specificity of 0.970 for screening, and an accuracy of 0.927 for staging [^20^].

Yang et al. reported an accuracy of 92.46% and an AUC of approximately 0.89 [^21^], whereas Devnath et al. achieved an accuracy of 92.68% [^22^]. Dong et al. found a mean AUC of 0.98, with values above 0.95 for the categories evaluated [^23^].

In another study, Devnath et al. used an ensemble model that obtained a sensitivity of 90.14%, specificity of 92.68%, accuracy of 91.50%, and F1 score of 90.78% [^24^].

For disease staging, Sun et al. [^25^] reported an accuracy of 90.4%, sensitivity of 78.4%, specificity of 95.6%, F1 score of 80.9%, and AUC of 0.96. The AUC for stage I was 0.89.

Using a ResNet-34-based model, Liu et al. [^26^] identified an accuracy of 89%, AUC of 0.98, and QWK of 0.94.

On the independent evaluation set, Li et al. [^27^] reported an accuracy of 95%, AUC of 0.947, and sensitivity of 100%.

For the MLANet model, Wang et al. [^28^] found an accuracy of 97.87%, 98.03%, and 95.40% in the internal, external, and prospective evaluations, respectively. For stage I classification, this model achieved an accuracy of 97.16%, recall of 98.25%, precision of 93.42%, and F1 score of 95.59%.

The performance metrics individually reported by the included studies are comparatively presented in Figure 2.

**Figure 2.**
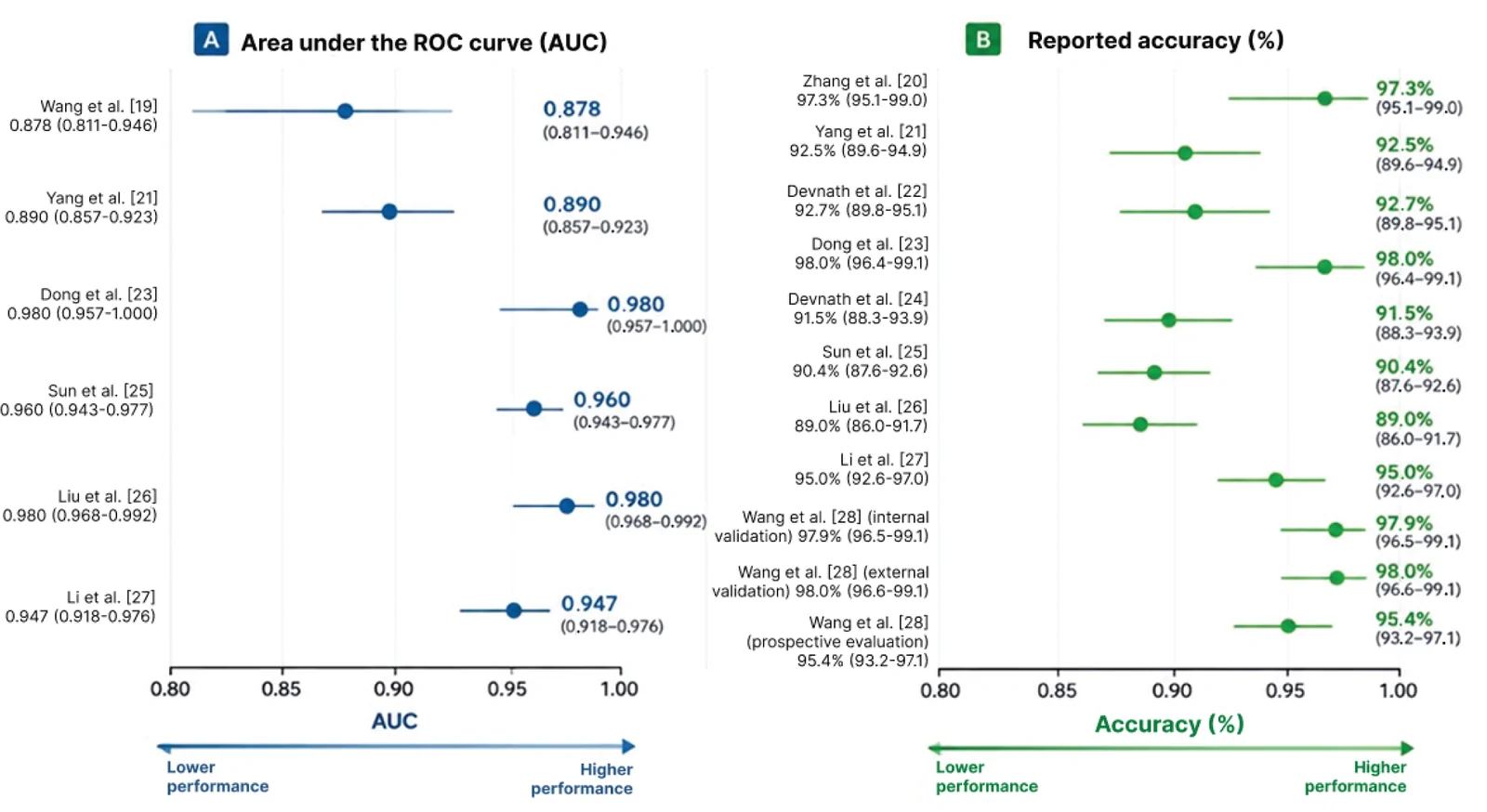
Comparison performance metrics reported in the included studies. ROC = received operating characteristic. Note: performance metrics are presented individually and do not constitute a meta-analysis. AUC and accuracy values were not pooled across studies because of heterogeneity in diagnostic tasks, populations, and validation designs. Panel A includes studies with verifiable AUC values. The study by Wang et al. [^19^] was not included in the accuracy panel because this metric was not reported in the publication. The study by Wang et al. [^28^] is shown separately for the internal, external, and prospective evaluations.

### COMPARISON WITH HUMAN READERS

Comparison between AI and human readers were available in three studies.

Wang et al. [^19^] compared the performance of Inception-V3 with that of two radiologists. The model achieved an AUC of 0.878, compared with 0.668 and 0.772 for the two radiologists, respectively.

Zhang et al. [^20^] also compared model performance with that of two groups of radiologists, who achieved accuracies of 0.87 and 0.84, respectively, for the staging task.

Wang et al. [^28^] compared the performance of the MLANet model with that of three radiologists during the prospective evaluation. It achieved an accuracy of 95.40%, recall of 93.72%, precision of 93.72%, and an F1 score of 93.72%, Radiologists 1, 2 and 3 achieved accuracies of 86.21%, 93.10%, and 96.55%; recall values of 74.68%, 92.21%, and 94.48%; precision values of 85.55%, 89.74%, and 96.01%; and F1 scores of 78.06%, 90.87%, and 95.21%, respectively.

In this prospective evaluation, the MLANet model obtained higher values than radiologists 1 and 2 across all four metrics, whereas radiologist 3 achieved higher scores than the AI model across all four metrics.

Thus, the results indicate that the MLANet model demonstrated competitive performance relative to the human readers evaluated, without demonstrating consistent superiority over all radiologists.

Previously reported average estimates for AI and radiologists were not used because these averages could not be reproducibly reconstructed from the included studies.

### VALIDATION AND GENERALIZABILITY

Validation strategies varied across studies. Yang et al. [^21^], Devnath et al. [^22^], Devnath et al. [^24^], Sun et al. [^25^], and Liu et al. [^26^] primarily used internal validation, cross-validation, or training/test splits, with no documented evidence of multicenter external validation.

Dong et al. [^23^] evaluated an observational cohort, with no documented evidence of independent external validation. Wang et al. [^19^] used a retrospective test set, which was not considered external validation.

Zhang et al. [^20^] assessed an independent test cohort, whereas Li et al. [^27^] used independent test data. These designs allowed performance to be assessed on data not directly used during training, although they differed from multicenter external validation.

Wang et al. [^28^] incorporated internal, external, and prospective evaluations, in which the MLANet model achieved accuracies of 97.87%, 98.03%, and 95.40%, respectively.

### INTERPRETABILITY AND CLINICAL INTEGRATION

More recent studies incorporated attention mechanisms and architectures designed to identify lesions, although the available evidence focused primarily on model development and evaluation.

The studies included did not provide sufficient evidence to determine the impact of these technologies on the efficiency of surveillance programs, interpretation times, or clinical outcomes, or to establish whether they could replace specialized radiologic assessment.

### TEMPORAL TRENDS

The 10 studies were published between 2020 and 2024. Over this period, methodological approaches evolved from CNN architectures primarily used for detection to more complex approaches incorporating segmentation, classification and staging, along with ensemble strategies and attention mechanisms.

Earlier studies employed architectures such as Inception-V3, U-Net, ResNet-34, and CheXNet, while late studies incorporated ensemble learning, EfficientNet, multistage models, and attention-based architectures, including a MLANet model.

The temporal evolution of the publications and methodologic approaches is shown in Figure 3.

**Figure 3.**
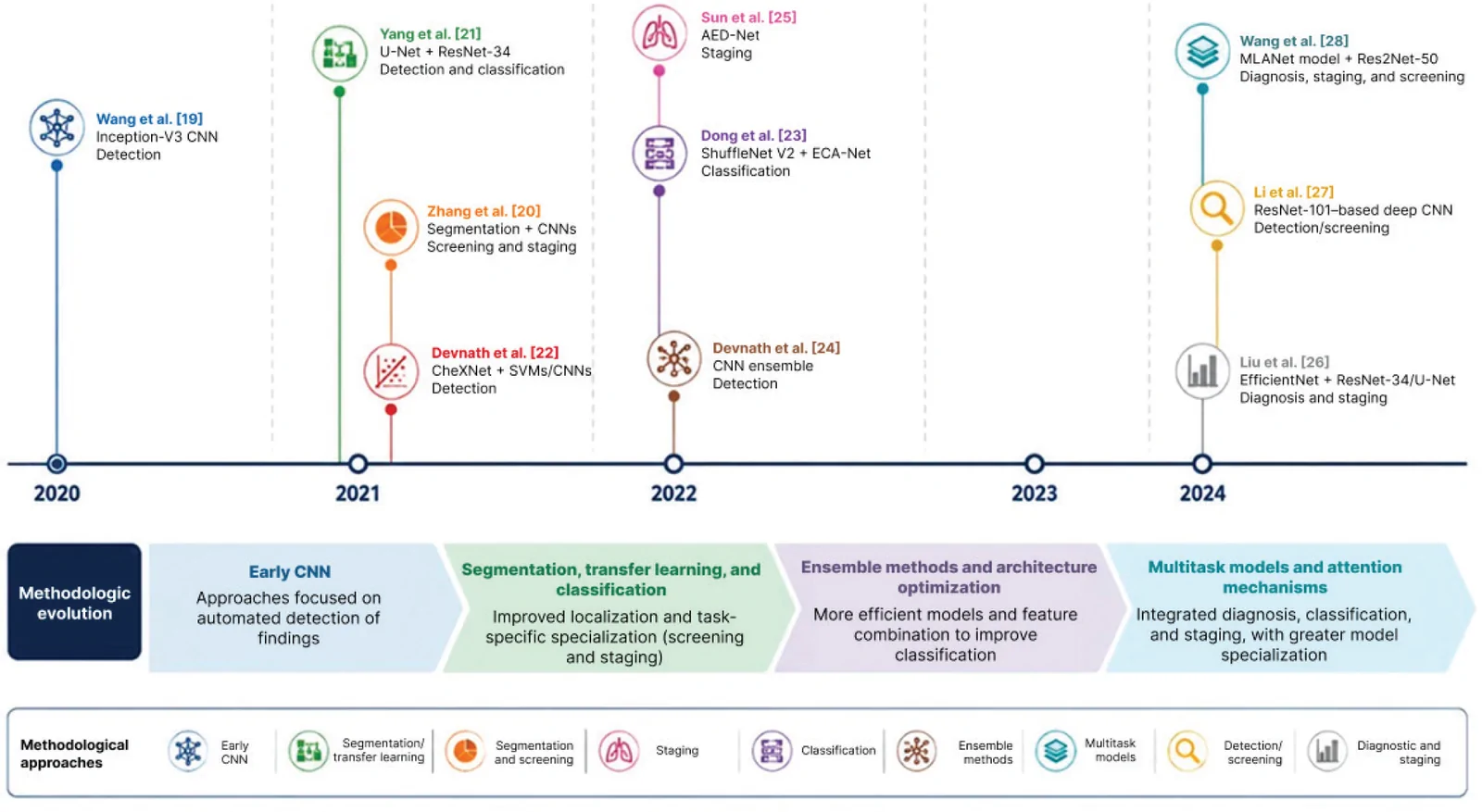
Timeline of publications and evolution of methodologic approaches. AED = Asymmetric EncoderDecoder Network; CNN = convolutional neural network; ECA = Efficient Attention Channel; MLANet = Multi-scale Lesion-aware Attention Networks; SVM = support vector machines. Note: the evolution of methodological approaches is presented descriptively and does not imply comparative superiority among the architectures.

### GEOGRAPHIC DISTRIBUTION

The included studies were conducted exclusively in China (n = 8) and Australia (n = 2), with a marked concentration of studies in China. The geographic distribution of the included studies is shown in Figure 4.

**Figure 4.**
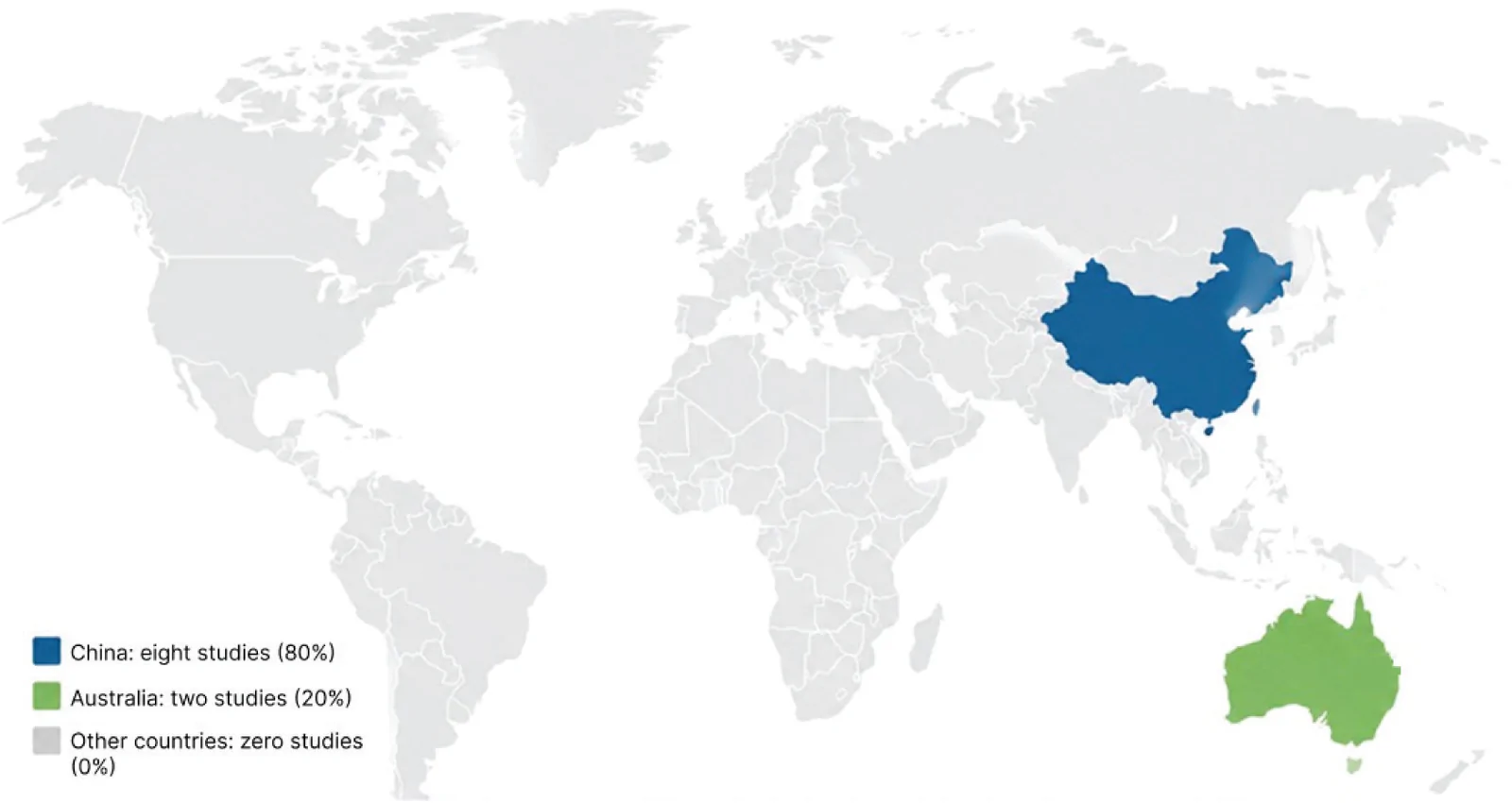
Geographic distribution of the studies included in the systematic review. Note: studies were assigned to the country of primary artificial intelligence model development or evaluation, as reported in each publication.

## Discussion

This systematic review synthesized the available evidence on the use of AI for the detection, classification, and staging of occupational pneumoconiosis using imaging techniques. The review included 10 studies published from 2020 to 2024 and conducted in China (n = 8) and Australia (n = 2), all of which were based on chest radiographs. Overall, conjunto, different deep learning models demonstrated favorable performance. However, considerable heterogeneity in study populations, diagnostic tasks, model architectures, reference standards, and validation strategies precludes deriving an overall estimate of AI performance from these findings.

### MAIN FINDINGS AND TECHNOLOGICAL EVOLUTION

CNNs were the predominant approach, encompassing architectures such as Inception-V3, ResNet, DenseNet, EfficientNet, CheXNet, and U-Net, as well as ensemble strategies and attention mechanisms. More recent studies incorporated multistage models and architectures designed for lesion identification, reflecting a transition from automated detection toward more complex classification and staging tasks.

Reported performance was generally high. Wang et al. [^19^] demonstrated an AUC of 0.878 (95% CI, 0.811-0.946), whereas Zhang et al. [^20^] obtained an accuracy of 0.973 for screening and 0.927 for staging. Yang et al. [^21^] reported an accuracy of 92.46% and an AUC of approximately 0.89. Other studies, including Dong et al. [^23^] y Liu et al. [^26^], showed an AUC of 0.98, although for different tasks and populations. Using a large dataset, Li et al. [^27^], found an AUC of 0.947 and an accuracy of 95% on an independent evaluation set. Wang et al. [^28^] achieved accuracies of 97.87%, 98.03%, and 95.40% in the internal, external, and prospective evaluations, respectively.

These findings support the potential role of AI as an assistive tool for automated chest radiograph analysis. However, performance metric derived from different tasks cannot be interpreted as directly equivalent; an AUC for detection, an accuracy for staging, and a metric derived from multiclass classification. Therefore, the available evidence does not permit a methodologically valid overall estimate of AI performance or conclusions regarding the superiority of any particular architecture.

### COMPARISON WITH HUMAN READERS

Comparisons with human readers were available in only some of the included studies and differed in terms of populations, diagnostic tasks, the number and experience of readers, and performance metrics. Wang et al. [^19^] reported an AUC of 0.878 for the model compared with 0.668 and 0.772 for the two radiologists evaluated. Zhang et al. [^20^] also compared model performance with that of two groups of radiologists, who achieved accuracies of 0.87 and 0.84, respectively, for staging.

In Wang et al. [^28^], the MLANet model was prospectively compared with three qualified radiologists and achieved an accuracy of 95.40%, compared with 86.21%, 93.10%, and 96.55% for the three readers, respectively. For recall, precision, and F1 score, the MLANet model obtained 93.72%, 93.72%, and 93.72%, respectively, whereas the readers showed varying values for each metric. The third radiologist achieved higher values than the model across all four reported metrics. Thus, these findings support the competitive performance of the MLANet model relative to specialized readers but do not establish the overall superiority of AI.

Collectively, the available evidence suggests that some AI models may achieve performance comparable to or even higher than that of certain human readers under specific evaluation conditions. However, the available comparisons are insufficient to establish the overall superiority of AI over radiologists. Reader experience, reference standard, case difficulty, and evaluation design may substantially influence the results.

### HETEROGENEITY AND GENERALIZABILITY

Heterogeneity is a major limitation to interpreting the available evidence, since the included studies addressed different forms of pneumoconiosis, populations with diverse occupational characteristics, and datasets of widely varying sizes. Moreover, the evaluated tasks included detection, screening, classification, and staging, with difference reference standards applied across the studies.

Such heterogeneity is particularly relevant when comparing models because a system trained to detect pneumoconiosis cannot necessarily be assessed using the same metrics or criteria as a model developed to distinguish radiologic stages, Individual performance metrics should therefore be interpreted within the methodological context of each study and should not be regarded as interchangeable measures of diagnostic performance.

Generalizability represents an additional concern, as several studies relied on internal data splits, cross-validation, or leave-one-out cross-validation. Although these strategies are useful for assessing performance within the available data, they are not equivalent to external validation.

Zhang et al. [^20^] and Li et al. [^27^] used independent evaluation sets; however, independence of the test does not necessarily constitute multicenter external validation.

Among the included studies, Wang et al. [^28^] used the most comprehensive evaluation strategy, incorporating internal, external, and prospective evaluations. This design provides stronger evidence regarding model generalizability than internal validation alone. Nevertheless, external or prospective evaluation by itself does not guarantee consistent performance across different occupational populations, radiographic equipment, acquisition protocols, or surveillance systems.

### RISK OF BIAS, TRANSPARENCY, AND REPRODUCIBILITY

Performance metrics should be interpreted alongside study methodological quality. PROBAST provides a structured assessment of participants, predictors, outcomes, and analysis, including risk of bias and concerns about applicability, whereas TRIPOD addresses the transparence and completeness of reporting.

High performance on a retrospective dataset does not necessarily indicate robustness to shifts in data distribution. Differences in demographic and occupational characteristics, disease prevalence, image quality, imaging equipment, and classification criteria may lead to data shift and affect model performance.

Therefore, reproducibility requires detailed reporting of participant and image selection, data partitioning, model architecture, training procedures, validation strategies, and evaluation metrics. Such transparency is essential for determining whether findings can be reproduced and whether model performance can generalize to independent settings.

### IMPLICATIONS FOR OCCUPATIONAL HEALTH

The findings have particular relevance for the surveillance of dust-exposed workers, given that all 10 included studies used chest radiography. With appropriate validation, such systems could potentially support screening, facilitate prioritization of radiographs for specialist review, assist radiographic classification, and enable the analysis of large volumes of imaging data.

These potential applications should not, however, be interpreted as evidence of established clinical utility. Before implementation in occupational surveillance programs, prospective multicenter evidence would be needed to determine reproducibility, performance across different populations and imaging equipment, interoperability, feasibility, costs, effects on clinical workflows, and safety.

Similarly, the use of attention mechanisms and architectures designed for lesion identification should not be regarded, in itself, as evidence of clinical interpretability. Clinical utility of these systems will depend not only on their performance metrics but also on the ability to understand their errors, implement appropriate mechanisms for human oversight, and clearly define professional accountability.

### PRIORITIES FOR FUTURE RESEARCH

The available evidence points to several priorities for future research. These include conducting multicenter studies across geographically diverse settings and occupational populations with different exposure profiles, strengthening independent external validation and prospective evaluation, and establishing more standardized criteria for case definition, image acquisition, radiographic classification, and performance assessment.

Future studies should provide sufficient information for transparent evaluation of risk of bias and reporting quality and to support model reproducibility. Research should also extend beyond performance metrics derived from retrospective evaluations to examine outcomes related to clinical utility, interpretation time, specialist referral, costs, implementation feasibility, and outcomes relevant to workers.

Finally, future research could also investigate models that integrate radiographic features with occupational information, such exposure history, duration, and intensity, and relevant clinical variables. Such integration could facilitate the development of decision-support systems better suited to occupational respiratory disease surveillance.

### STRENGTHS AND LIMITATIONS

A strength of this review is the explicit distinction between internal validation, cross-validation, independent test sets, external validation, and prospective evaluation, which allows for a more precise interpretation of the available evidence regarding model generalizability.

The main limitations included the small number of studies, their geographic concentration in China and Australia, and considerable heterogeneity in study populations, diagnostic tasks, model architectures, and validation strategies. Moreover, comparisons with human readers were limited and heterogeneous across studies.

Insufficient comparability of diagnostic tasks and evaluation designs also precluded meta-analysis and the calculation of pooled performance estimates. Nevertheless, the findings should be interpreted as a critical narrative synthesis rather than as a meta-analytic estimate of AI diagnostic performance.

### OVERALL INTERPRETATION

Taken together, the available evidence suggests that AI has considerable potential to support the detection, classification, and staging of occupational pneumoconioses using chest radiography, with several studies reporting high performance [^25^]. However, the available evidence remains limited by methodological heterogeneity, the geographic concentration of the studies, uneven availability of external validation and prospective evaluation, and limited evidence of clinical utility under real-world conditions.

Therefore, the main challenge in this field is no longer simply to achieve higher performance metrics on development datasets, but to demonstrate that such performance is reproducible, safe, and clinically useful across independent and diverse occupational populations. Based on the current evidence, AI should be considered a complementary tool to support occupational surveillance and specialist interpretation rather than a substitute for clinical and radiologic assessment by qualified professionals [^29^].

## Conclusions

This systematic review indicates that AI, particularly deep learning models and CNNs, has considerable potential to support the detection, classification, and staging of occupational pneumoconioses using chest radiography. The 10 included studies, published between 2020 and 2024, reflect a shift toward more complex architectures, including ensemble models, multistage strategies, and attention mechanisms, with favorable performance across different diagnostic tasks.

However, the available evidence does not support deriving an overall estimate of diagnostic performance or establishing the general superiority of AI over radiologists. Differences in populations, diseases, diagnostic tasks, sample sizes, reference standards, model architectures, and validation strategies preclude direct comparisons and quantitative synthesis of the metrics reported.

Methodological heterogeneity, geographic concentration of the studies, and limited external validation and prospective evaluation represent major barriers to determining the generalizability of these systems, particularly because internal validation and cross-validation should not be considered equivalent to independent external validation. Although Wang et al. [^28^] included internal, external, and prospective evaluation, their findings require independent replication across diverse occupational populations and geographic contexts.

Therefore, AI should be currently regarded as complementary tool to support screening, radiographic interpretation, and classification of occupational pneumoconioses rather than a substitute for specialized clinical and radiologic assessment. Its incorporation into surveillance programs for dust-exposed workers will require further evidence of reproducibility, generalizability, clinical utility, feasibility, and impact on real-world conditions.

Future research should prioritize multicenter, prospective, and geographically diverse studies with standardized protocols for image acquisition and radiographic classification, transparent reporting, independent external validation, and assessment of clinical and occupational outcomes. Considerations for implementation, such as costs, interoperability, equity, regulation, professional accountability, and human oversight, should also be addressed.

Taken together, the evidence supports the potential role of AI as a complementary component of future strategies for the surveillance and detection of occupational pneumoconioses but indicates that the application of AI in this field remains in transition from model validation to clinical and occupational implementation. The main challenge is not merely to achieve high diagnostic performance but also to demonstrate that AI models can perform safely, reproducibly, and equitably while providing verifiable clinical and occupational benefit for workers exposed to silica and other mineral dusts.

## Data Availability

Upon publication, the data will be made available by the authors upon request.
